# To: Using the 2026 Surviving Sepsis Campaign Guidelines in Practice

**DOI:** 10.62675/2965-2774.20260148

**Published:** 2026-07-21

**Authors:** Rodolpho Augusto de Moura Pedro, Luiz Marcelo Sá Malbouisson

**Affiliations:** 1 Universidade de São Paulo Department of Gastroenterology Faculdade de Medicina São Paulo SP Brazil Liver and Gastroenterology Critical Care Unit, Department of Gastroenterology, Faculdade de Medicina, Universidade de São Paulo - São Paulo (SP), Brazil.

**DEAR EDITOR**

We read with interest the article "Using the 2026 Surviving Sepsis Campaign (SSC) Guidelines in Practice" by Azevedo et al.^(1)^ We aim to complement this discussion by presenting an additional controversy regarding albumin administration in patients with cirrhosis.

While the 2021 Surviving Sepsis Campaign (SSC) document made only a passing reference to "cirrhosis", without providing specific guidance,^(2)^ the 2026 guidelines^(3)^ now introduce a specific recommendation for patients with end-stage liver disease: "for patients with cirrhosis, use of albumin in addition to crystalloids may be preferred".

We recognize that part of the challenge in addressing such a specific population lies in the fact that patients with cirrhosis are frequently excluded from major clinical trials,^(4)^ partly due to stigma, and partly due to particular physiological features such as prolonged capillary refill time and baseline hyperlactatemia.

Nonetheless, given the wide dissemination and influence of this guideline, we believe that certain contextual considerations are essential for intensivists caring for patients with cirrhosis. The recommendation appears to rely primarily on the FRISC trial,^(5)^ a single-center study published in 2021 comparing 5% albumin (250mL bolus followed by 50mL/hour) *versus* normal saline for fluid resuscitation in cirrhotic patients with sepsis-related hypotension. In that trial, the albumin group demonstrated a higher rate of early hypotension reversal, a greater reduction in lactate levels over the same period, and possibly lower mortality as a secondary outcome.

However, the same group subsequently published the ALPS trial^(6)^ in 2022, in which 20% albumin (0.5-1g/kg) was compared with normal saline in a similar clinical setting. Although the findings of faster hypotension resolution and lactate reduction were reproduced, no mortality benefit was observed. More importantly, 22% of patients in the albumin group required discontinuation of therapy due to serious adverse events, most commonly respiratory failure, accompanied by a mean reduction of nearly 50 points in the ratio between the partial pressure of arterial oxygen and the fraction of inspired oxygen (PaO_2_/FiO_2_) ratio, a point highly relevant in the context of pulmonary sepsis with respiratory failure.

Additional considerations should also be acknowledged. In this population, using total body weight to guide fluid dosing may be misleading, as ascites can lead to overestimation and potential overdosing. Furthermore, there is no clearly established equivalence between albumin and crystalloid dosing, raising the possibility that one group may have received greater effective volume expansion than the other. Diagnosis certainty is also crucial, as hypotension in cirrhosis may in fact result from variceal bleeding, a scenario in which liberal infusion could increase the portal pressure gradient and potentially precipitate rebleeding. Finally, cost remains a critical issue in many settings. In our public hospital, located in a low- and middle-income country, a vial of 20% albumin might cost $20,7, whereas 1L of crystalloid solution costs around $0.54.^(7)^

In conclusion, addressing patients with cirrhosis in the new guideline represents an important and welcome advance. However, greater clarity regarding albumin concentration, dosing, and the need for vigilant monitoring of adverse effects is essential to ensure its safe and effective use.
